# The TMS Motor Map Does Not Change Following a Single Session of Mirror Training Either with Or without Motor Imagery

**DOI:** 10.3389/fnhum.2017.00601

**Published:** 2017-12-12

**Authors:** Mark van de Ruit, Michael J. Grey

**Affiliations:** ^1^Neuromuscular Control Laboratory, Department of Biomechanical Engineering, Delft University of Technology, Delft, Netherlands; ^2^Acquired Brain Injury Rehabilitation Alliance, School of Health Sciences, University of East Anglia, Norwich, United Kingdom

**Keywords:** mapping, TMS, motor imagery, motor learning, action observation, mirror training

## Abstract

Both motor imagery and mirror training have been used in motor rehabilitation settings to promote skill learning and plasticity. As motor imagery and mirror training are suggested to be closely linked, it was hypothesized that mirror training augmented by motor imagery would increase corticospinal excitability (CSE) significantly compared to mirror training alone. Forty-four participants were split over two experimental groups. Each participant visited the laboratory once to receive either mirror training alone or mirror training augmented with layered stimulus response training (LSRT), a type of motor imagery training. Participants performed 16 min of mirror training, making repetitive grasping movements paced by a metronome. Transcranial magnetic stimulation (TMS) mapping was performed before and after the mirror training to test for changes in CSE of the untrained hand. Self-reports suggested that the imagery training was effective in helping the participant to perform the mirror training task as instructed. Nonetheless, neither training type resulted in a significant change of TMS map area, nor was there an interaction between the groups. The results from the study revealed no effect of a single session of 16 min of either mirror training or mirror training enhanced by imagery on TMS map area. Despite the negative result of the present experiment, this does not suggest that either motor imagery or mirror training might be ineffective as a rehabilitation therapy. Further study is required to allow disentangling the role of imagery and action observation in mirror training so that mirror training can be further tailored to the individual according to their abilities.

## Introduction

Mirror training was successfully introduced in the 1990s to alleviate phantom limb pain in amputees (Ramachandran and Rogers-Ramachandran, 1996). In mirror training a mirror is placed in front of the participant in a parasaggital plane with each limb, e.g., hand or foot, positioned on either side of the mirror. When one limb is moved whilst watching its mirror reflection, the visual illusion is created that the passive limb behind the mirror is moving. With repetitive practice this has been found to lead topographic reorganization of the somatosensory cortex, in the hemisphere associated with the amputated limb (for review Ramachandran and Altschuler, 2009). In healthy participants, an increase in corticospinal excitability (CSE), expressed as a significant change in motor evoked potential (MEP) amplitude, following mirror training has been linked to improved motor performance of the non-trained hand after just 10 sets of 30 s on a ball rotation task (Nojima et al., 2012).

The positive outcomes associated with mirror training have been attributed to its close correspondence to motor imagery and action observation (Stevens and Stoykov, 2003; Vogt et al., 2013). Motor imagery is the process of mentally rehearsing a movement without creating any overt motor output, and has been reported to be able to lead to improved motor performance (Feltz and Landers, 1983; Williams et al., 2013). Similarly for action observation, motor performance has been found to improve by observing a movement performed by another individual (Vogt, 1995; Vogt and Thomaschke, 2007). Whereas, physical practice has long been considered essential in motor learning and rehabilitation (e.g., Butefisch et al., 1995), these findings might suggest imagery and action observation are valuable complements.

The potential of mirror training, motor imagery and action observation in motor learning and rehabilitation can be explained by the finding of shared neural representations between motor execution, motor imagery and action observation (Jeannerod, 1994; Rizzolatti et al., 1996; Grezes and Decety, 2001; Guo et al., 2016; Kilintari et al., 2016). These findings have been supported by the evidence from transcranial magnetic stimulation (TMS) studies that demonstrated both motor imagery and action observation result in an increase in MEP amplitude in healthy participants. This suggests engagement of motor cortical areas during these passive processes (Fadiga et al., 1995; Kasai et al., 1997; Stinear and Byblow, 2003; Lepage et al., 2010; Roosink and Zijdewind, 2010; Naish and Obhi, 2015). These studies reported an increase in MEP amplitude specific to the muscle involved for, and in time with, the imagined or observed movement. Moreover, MEP amplitude is significantly increased when combining action observation and motor imagery compared to when applying them individually (Wright et al., 2014, 2016). In addition to the MEP amplitude the TMS map area is also increased during and following imagery (Pascual-Leone et al., 1995; Cicinelli et al., 2006; Marconi et al., 2007). Maps are obtained by recording MEPs in response to TMS stimuli delivered at constant intensity but different sites over the motor cortex. The position and MEP data are then merged to produce topographical map representing CSE across the cortex (Wassermann et al., 1992). Maps are quantified by calculating the area of excitability beyond a minimal threshold. In conclusion both TMS map area as well as MEP amplitude can be used to quantify changes in CSE following motor imagery and action observation.

Increased MEP amplitude for healthy participants has also been reported following mirror training, both during and immediately after the training (Garry et al., 2005; Fukumura et al., 2007; Funase et al., 2007; Kang et al., 2011; Nojima et al., 2012). This would suggest that if motor imagery and action observation play an important role in mirror training, either could be used to increase CSE following mirror training alone. This idea is supported by the finding that greater increases in MEP amplitude are found in participants with better imagery ability (Williams et al., 2012). Moreover, improved imagery ability will benefit the learning process by recruitment of different brain regions (Guillot et al., 2008). It has recently been demonstrated that imagery can be improved by gradually building the complexity of the image, a technique known as Layered Stimulus and Response Training (LSRT) (Williams et al., 2013). In LSRT, image detail is gradually introduced using a layered approach based on the participants feedback (Cumming et al., 2017). As a result, the aim of LSRT is to improve imagery quality and ability in a short space of time.

The primary aim of the study was to determine if a single session of LSRT in addition to mirror training would induce significantly greater changes in TMS map area than mirror training alone. Previous studies using TMS to assess CSE have used stimuli at a single intensity (Garry et al., 2005; Fukumura et al., 2007; Kang et al., 2011; Reissig et al., 2015; Grunt et al., 2017) or stimulus-response curves (Avanzino et al., 2014; Ruddy et al., 2016). Here we used a rapid mapping technique (van de Ruit et al., 2015) in order to detect changes in the distribution of excitability and uneven expansions of the cortical map, whilst measuring changes in CSE similar to other single-pulse TMS techniques (Ridding and Rothwell, 1997). Specifically, we tested the hypothesis that motor imagery training in addition to mirror training would produce a greater map area (i.e., increased excitability) compared to mirror training alone.

## Materials and methods

### Participants

Forty-four healthy participants (21 ± 4 year, range 18–35, 28 female) were recruited for the study. Informed consent was obtained from all individual participants included in the study. Participants were screened for contra-indications to TMS using a modified version of the TMS adult safety questionnaire originally suggested by Keel et al. (2001). No participants reported any neurological impairments or illness. The study was approved by the University of Birmingham's Science, Technology, Engineering and Mathematics ethics committee (ERN_13-0701), and all experiments were performed in accordance with the Declaration of Helsinki.

### Electromyography

Bipolar surface electrodes (Blue Sensor N, Ambu, Denmark) were used to record the electromyographic (EMG) activity from the finger extensor muscle of the non-dominant hand, extensor digitorum communis (EDC), which was the primary muscle of interest. At the same time, EMG was recorded from the finger flexor muscle, flexor digitorum superficialis (FDS). Skin preparation and electrode placement were performed as per SENIAM guidelines (Hermens et al., 2000). All EMG signals were amplified (EDC; 2k, FDS; 5k), band pass filtered (20–1,000 Hz), and digitally sampled at 5 kHz to be stored for offline analysis.

### Transcranial magnetic stimulation

Magnetic stimulation was delivered by a Magstim Rapid^2^ (Magstim Ltd, Dyfed, United Kingdom) with a custom made polyurethane coated 90 mm “batwing” shaped figure-of-8 coil. The coil was always held in a 45° angle to the midline with the handle pointing backward, so to induce a current in the brain flowing in postero-anterior direction (Brasil-Neto et al., 1992). The “hotspot,” or stimulation site resulting in the largest motor evoked potentials (MEPs), was found by visual inspection of the EMG recorded from the contralateral EDC muscle. The hotspot was used to determine the resting motor threshold (RMT). Starting at 60% maximum stimulator output, the threshold intensity was defined by the intensity at which at least 5 out of 10 stimuli evoked MEPs with a peak-to-peak amplitude > 50 μV (Rossini et al., 1994; Groppa et al., 2012). Coil position and orientation was manually determined and monitored using frameless stereotaxy (BrainSight 2, Rogue Research Inc., Montreal, Canada). To obtain TMS maps stimuli were delivered at 120% RMT.

### Peripheral nerve stimulation (PNS)

Motor evoked potentials (MEPs) were normalized to the electrically evoked maximal M-wave (M_max_) to ensure valid comparison between participants. To obtain the M_max_ a bipolar probe was used to stimulate the deep radial nerve EDC and ulnar nerve FDS of the non-dominant hand at the level of the elbow using a constant current stimulator (Digitimer DS7A, Digitimer Ltd, Welwyn Garden City, UK). M_max_ is obtained by applying a few stimuli to the peripheral nerve with successively larger stimulation current until a supramaximal current produces the largest M-wave. This ensures we find the M_max_ quickly without administering too many, uncomfortable, stimuli (Palmieri et al., 2004).

### Experimental protocol

**Figure 1:**
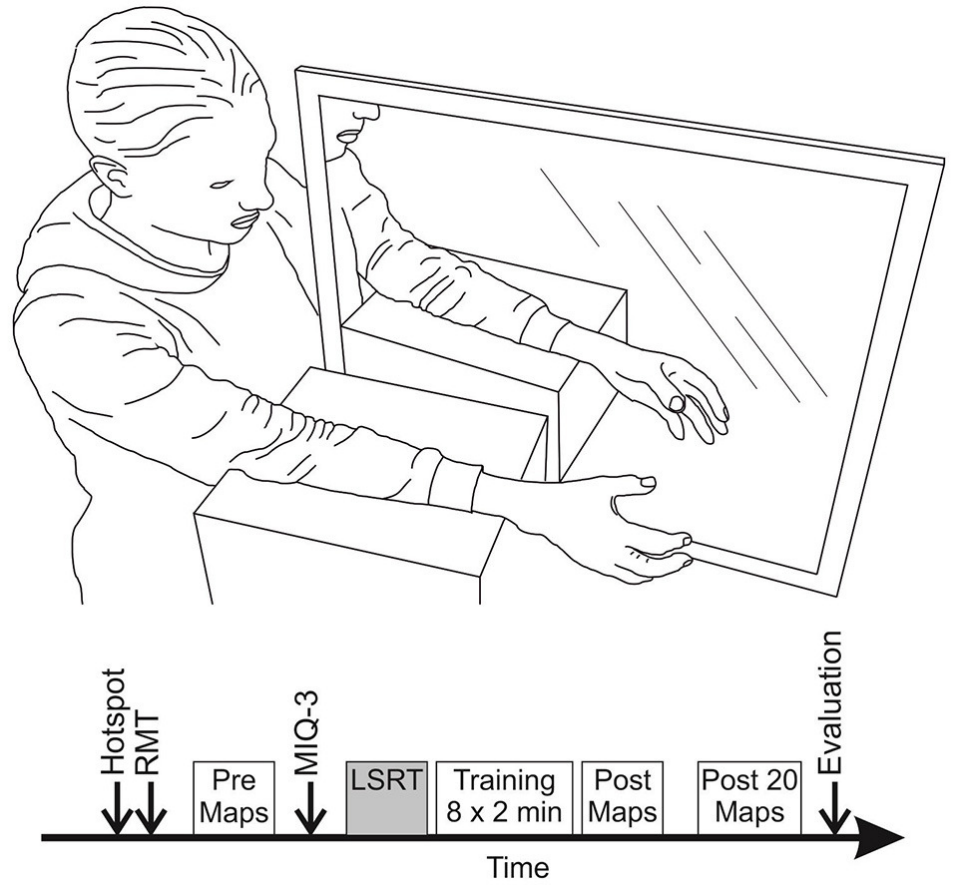
Overview of the experimental set-up and protocol during mirror training. **(Top)**: Participants were seated comfortably with their arms supported by foam blocks and lined up, one on each side of the mirror. **(Bottom)**: Each session was started with finding the hotspot and resting motor threshold (RMT) for the EDC muscle. Then data for three TMS maps was collected. Imagery ability was assessed of all participants using the MIQ-3 questionnaire. Participants assigned to the imagery group received ~10 min of layered stimulus response training (LSRT) while the control group went straight through to the mirror training. Mirror training was done in 8 blocks of 2 min with 15 s breaks. After mirror training data for three TMS maps was collected directly and 20 min after end of the training. The session was finished with an evaluation questionnaire.

During TMS assessments participants were seated comfortably in a custom-built chair with their non-dominant arm resting pronated on a height-adjustable stool. Participants' handedness was assessed using a modified version of the Edinburgh Handedness Inventory (Oldfield, 1971). The score runs from −100 to +100, indicating purely left or right handed respectively, with participants with a score between −40 and 40 classified as ambidextrous. The non-dominant hand was studied as associated hemisphere has been reported to be more sensitive to imagery and action observation induced changes in MEP amplitude (Bianco et al., 2012). Three TMS maps were acquired (taking in total ~10 min) before and directly after mirror training with the muscles at rest. Another three TMS maps were acquired 20 min after the mirror training had finished. The latter was done in light of the finding that as some consolidation time is important before remodeling of neural pathways can be identified (Pascual-Leone et al., 2005). Before the start of the experiment, every participant was randomly assigned to the control or imagery training group. The *control group* only received mirror training, whereas the *imagery group* received additional imagery training prior to the mirror training. In both groups imagery ability was assessed using the movement imagery questionnaire (MIQ-3) (Williams et al., 2012). Following the assessment of imagery ability, the imagery training group received about 10 min of LSRT including explanation of the mirror training and imagery concepts. The control group received the same explanation, limited to how to perform mirror training task, before the training commenced. The time between the baseline TMS assessment and mirror training was equal for both groups. All participants received 8 blocks of 2 min (16 min in total) mirror training separated by 15 s rest breaks. A total of 16 min of training was adopted as a trade-off between training time adopted in other studies on the one hand and to avoid loss of attention on the other hand. Five minutes of mirror training was found sufficient to result in changes in MEP amplitude in one study (Nojima et al., 2012) but 10 min not enough in another study (Avanzino et al., 2014). Byblow et al. (2012) showed 20 min of bimanual passive wrist movement results in an increase in MEP amplitude, whereas different motor learning studies used 12, 16, or 30 min of training (Jensen et al., 2005; McAllister et al., 2011; Willerslev-Olsen et al., 2011). Moreover, pilot testing revealed more than 20 min of training was unfeasible given participants did get distracted and lost focus. Figure 1 provides an overview of the experimental setup and protocol.

#### Creating the TMS map

Corticospinal excitability (CSE) was assessed using TMS maps acquired using a novel rapid mapping procedure that takes advantage of frameless stereotaxy together with reduced interstimulus interval (van de Ruit et al., 2015). After finding the motor hotspot and motor threshold of the EDC muscle a square 6 × 6 cm grid was positioned over the hemisphere corresponding to the non-dominant hand. The grid was aligned such that the motor hotspot would be central in the map to ensure full mapping of the cortical representation was possible. For each TMS map, 80 stimuli were delivered at 120% of EDC RMT to pseudorandom locations in the square grid with each site receiving a single stimulus. Interstimuls interval (ISI) was set to 1.5 s as this frequency of stimulus delivery together with the small number of stimuli required for the protocol does not depress cortical excitability (Mathias et al., 2014) as has been shown with longer protocols. Each stimulus was applied at a pseudorandom location within the grid whilst being delivered at a different location than the previous stimulation.

#### Movement imagery ability

General motor imagery ability of simple movements was assessed using the Movement Imagery Questionnaire (MIQ-3; Williams et al., 2012). The MIQ-3 assesses the participants' visual and kinaesthetic imagery (KI) ability, and provides a measure for the ease with which participants generate the image. The visual imagery ability is divided up in the ability to image in an internal (first person; II) and external (third person; EI) perspective. Four movements (knee raise, jump, arm movement and waist bend) are performed and imaged three times, once for each imagery perspective. First, the movement is clearly explained and physically performed before the participant is instructed to imagine the movement from either a visual internal, visual external or kinaesthetic perspective. When the participants finished the imagery exercise, they were asked to rate the ease of imaging on a 7-point scale (1: very hard to see/feel−7: very easy to see/feel).

#### Layered stimulus response training

Participants assigned to the Imagery training group received ~10 min of Layered Stimulus Response Training (LSRT) to make their image as realistic and vivid as possible (Cumming et al., 2017). This training took place in the same environment and with the same body posture and arm position as in which the mirror training took place. Participants rested one arm on each side of the mirror in a foam support. Hands remained in a neutral position with the thumbs up throughout training. With their eyes closed, participants were first instructed to perform repetitive grasping movements with both hands; gently opening and closing the hand without making a tight fist or over-extending the fingers. These movements were performed for 30 s and were paced at 40 bpm with a metronome; the hand being either fully opened or closed at every beat. After this initial practice, participants were asked to describe the feelings in their hands and arms in as much detail as possible. Subsequently, participants were asked to keep both hands still and use the sensation that was easiest to identify during imaging the non-dominant hand making repetitive grasping movements. The image was then built by including more detail step-by-step, making sure to incorporate sufficient detail to benefit the imagery process but without overloading the participant with instructions (Williams et al., 2013). In the next step, movement of the dominant hand was introduced and eyes were kept open to allow using the mirror reflection to further facilitate the imagery. Following, the introduction of movement with the dominant hand, vividness of the imagery was checked, performing extra training following the LSRT format in case the imagery quality had deteriorated, which was based on verbal feedback by the participants.

#### Mirror training

In total, 16 min of mirror training was performed, divided in 8 blocks of 2 min and separated by 15 s rest breaks. The participant was instructed to keep the non-dominant hand still whilst making the repetitive mimed grasping movement with just the dominant hand. Participants in the imagery group were encouraged to imagine the non-dominant hand moving, using the mirror visual feedback to strengthen the illusion. They were encouraged to use the feelings and sensations practiced, thereby emphasizing using KI:

“*Now, imagine your right/left hand moving whilst watching the mirror reflection of your repetitively grasping left/right hand. Keep the hand behind the mirror still but imagine it is moving; using the mirror reflection to help you feel and see your left/right hand making the grasping movements*.”

For the control group, the verb “imagine” in this instruction was replaced by “see.” During the breaks participant were requested to keep both hands relaxed and still, and to remain focussed on the mirror reflection. In addition, participants in the imagery group were reminded to focus on the feelings and sensations whilst imaging.

#### Post training evaluation questionnaire

In order to assess the task compliance and engagement of all participants, they were asked to fill out a task evaluation questionnaire (equivalent to questionnaire used in Williams et al., 2013). To check task compliance, participants were asked to what extent they were engaged in the imagery process, if their imagery did get better throughout the training blocks and to what extent they performed the task as instructed and practiced. Moreover, participants were asked how easy they found it to image the feelings associated with the grasping movement of the non-dominant hand and how clear and vivid the image was that they could create. Finally, it was confirmed if participants had been aware of any physical movement in the non-dominant arm throughout the training. The first question was rated on a percentage scale (0%: not engaged, 100%; fully engaged) while all other questions were rated on a 7-point scale ranging from 1 (not at all/very hard/no image at all) to 7 (considerably/exactly/very easy/perfectly clear and vivid/full grasping).

### Data analysis

#### TMS maps

To create the TMS map data was analyzed offline with a bespoke MATLAB script (MATLAB Release 2012b, The MathWorks, Inc., Natick, Massachusetts, United States). All stimulation positions were projected in a 2D plane. Accordingly, each position was matched with its corresponding normalized MEP peak-to-peak (MEP_pp_) value as extracted from the EMG in a window 20–50 ms after stimulation.

Individual stimuli within a map were excluded from analysis when for the stimulation or corresponding MEP: (1) the root mean square value of the background EMG (50–5 ms before stimulation) was outside Mean ± 2 SD of all stimuli or peak-to-peak amplitude > 30 μV; (2) stimulation fell more than 10 mm outside the grid's border; (3) MEP size was larger than Mean ± 3.5 SD of all MEPs in the map; (4) angle and translation of the stimulus location fell outside the 99% predication interval of all stimuli.

Maps were quantified by the map area and center of gravity (COG). MEP_pp_ values were used to approximate a 6 × 6 cm grid composed of 2,500 pixels using MATLAB's “gridfit” function (D'Errico, 2005). The number of pixels with an approximated MEP_pp_ amplitude >10% of the maximum MEP_pp_ value, or >100 μV peak-to-peak if the 10% threshold was smaller than this, was calculated and expressed as total map area (in mm^2^). The maps COG x- and y-coordinate was calculated by using the MEP_pp_ amplitude and its position on the map, creating an amplitude weighted mean of the map. To quantify changes in the position of the COG, the x- and yCOG translation was calculated by subtracting the x- and y-position of the baseline map from map COG after mirror training (Δx and Δy). In this way, a negative value would indicate a shift in anterior (x) or lateral (y) direction and a positive value a shift posteriorly (x) or medially (y).

The absolute displacement was calculated by taking the Euclidian Distance (ED) between COGs of the median maps before and after the mirror training:

$$\begin{array}{l} ED=\;\sqrt{{\left(yCOG_{pre}-yCOG_{post}\right)}^{2}+{\left(xCOG_{pre}-xCOG_{post}\right)}^{2}} \end{array}$$

Full details of this process are provided in van de Ruit et al. (2015).

#### Imagery ability and post training evaluation questionnaire

Imagery ability is quantified for the different perspectives by taking the mean of the scores provided for each movement imaged using a internal, external or kinaesthetic perspective.

### Statistical analysis

Statistical testing was conducted with IBM SPSS Statistics 21. Tests were considered significant at α = 0.05. It was confirmed that the data did not violate any of the statistical tests assumptions. When the assumption of covariance matrix circularity was violated a Geisser–Greenhouse adjustment was made (denoted by GG following the *F*-test). Data are reported as Mean ± 1 SD unless otherwise noted.

#### TMS maps

To assess training induced changes in excitability, TMS map area was studied. First the map with the median map area at each time point (Pre, Post, Post20) was selected. In case only two baseline measures were considered, e.g., data was missing for one map, the map with the lowest mean background EMG was taken as the median. Subsequently, the difference in map area between the pre and post measurement was taken (Δarea = area_POST_–area_PRE_). A negative Δarea would thereby indicate a decrease in map area, and a positive difference an increase. The Δarea data was then tested for statistical significance with respect to the fixed value of 0 using a one sample *t*-test for both time points (Post, Post20) and groups (Control, Imagery), which would highlight an effect of mirror training on TMS map area. Further, a mixed design ANOVA (within factor: time point, between factor: group) was used to reveal any effects between groups and an effect of the consolidation time following training. The same analysis was performed for the translation of the COG (Δx and Δy).

To quantify the response rate to mirror training participants' were classified as positive responders, negative responders, or non-responders based on the change in map area. The three TMS maps acquired at baseline were used to quantify the baseline variance (standard deviation, SD) in map area. Participants were then classified based on the post training change in map area as positive responders (Δarea > 1 SD), negative responders (Δarea < 1 SD), and non-responders (Δarea within 1 SD).

#### Imagery ability and post training evaluation questionnaire

Imagery ability and scores to each question of the evaluation questionnaire were compared between the groups using an independent samples *t*-test.

#### Predicting factors of response to mirror training

A two-way multivariate analysis of covariance (2w-MANCOVA), with the dependent variables TMS map area at time points Post and Post20, was used to investigate whether time of the day the experiment was performed, baseline imagery ability and participants' experiences during the mirror training could predict participants' their response, i.e., change in map area. Both group and time of the day were used as fixed factors with imagery ability (KI, II and EI) and participants' responses to the post evaluation questionnaires (5 questions on 7-point Likert Scale) were included as covariates. The time of the day the experiment was performed was classified as 1–4 based on the following windows 08.00–11.00 (1), 11.00–14.00 (2), 14.00–17.00 (3), and 17.00–20.00 (4).

## Results

Data of 40 participants was analyzed, 20 in both groups. Data of four participants, two in both groups, had to be discarded as these data sets were incomplete as consequence of equipment malfunctioning. Scores for the Edinburgh Handedness Inventory indicated 36 participants were right handed (score: 90 ± 28). All other participants were left handed (score: −79 ± 22). Data for the FDS muscle was not analyzed, as for most participants the stimulation intensity used to acquire the data for the TMS maps was below motor threshold.

### General imagery ability

Baseline imagery ability was similar for both groups for each imagery perspective [KI: *t*_(38)_ = 0.13, *P* = 0.90; II: *t*_(38)_ = −0.70, *P* = 0.49; EI: *t*_(38)_ = 0.91, *P* = 0.37], usually rated between 5 and 6 (somewhat easy to feel/see–easy to feel/see).

### Layered stimulus response training

Following the initial practice of the movement with eyes closed, most participants reported specific feelings and sensations involving hand and arm movements. Seven participants could not identify any specific feeling and/or sensations. The most common feelings described were: “the muscles in the forearm contracting,” “the finger tips touching the palm when closing the hand” and “stretching of the skin between the fingers when opening the hand.” As these three feelings were commonly described, they were used by most participants to build the image. Most participants needed two or three practice runs to build the image of the closing and opening hand behind the mirror whilst keeping the eyes closed and both hands still. Another two or three practice runs were needed using the mirror reflection of the moving, dominant, hand to further improve the imagery. Participants reported improvement of image quality throughout the imagery training. Many participants reported that introduction of movement of the dominant hand and using the mirror visual feedback lead to difficulties with the imaging process. Participants reported verbally how imagery improved with extra practice runs, of which usually two or three were required.

### TMS maps

In order to study the effect of mirror training with or without LSRT on CSE the COG and area of the TMS map were examined. The maps included in the analysis for all sessions were constructed out of 73 ± 2 stimuli. The SD of the Δarea in the baseline recordings (with respect to the median map) was 265 mm^2^ for the imagery group and 281 mm^2^ for the control group. Therefore, a participant was defined as a positive responder if Δarea > 281 mm^2^ (control group) and Δarea > 265 mm^2^ (imagery group), a negative responder if Δarea < 281 mm^2^ (control group) and Δarea < 265 mm^2^ (imagery group) and a non-responder in all other cases.

**Figure 2:**
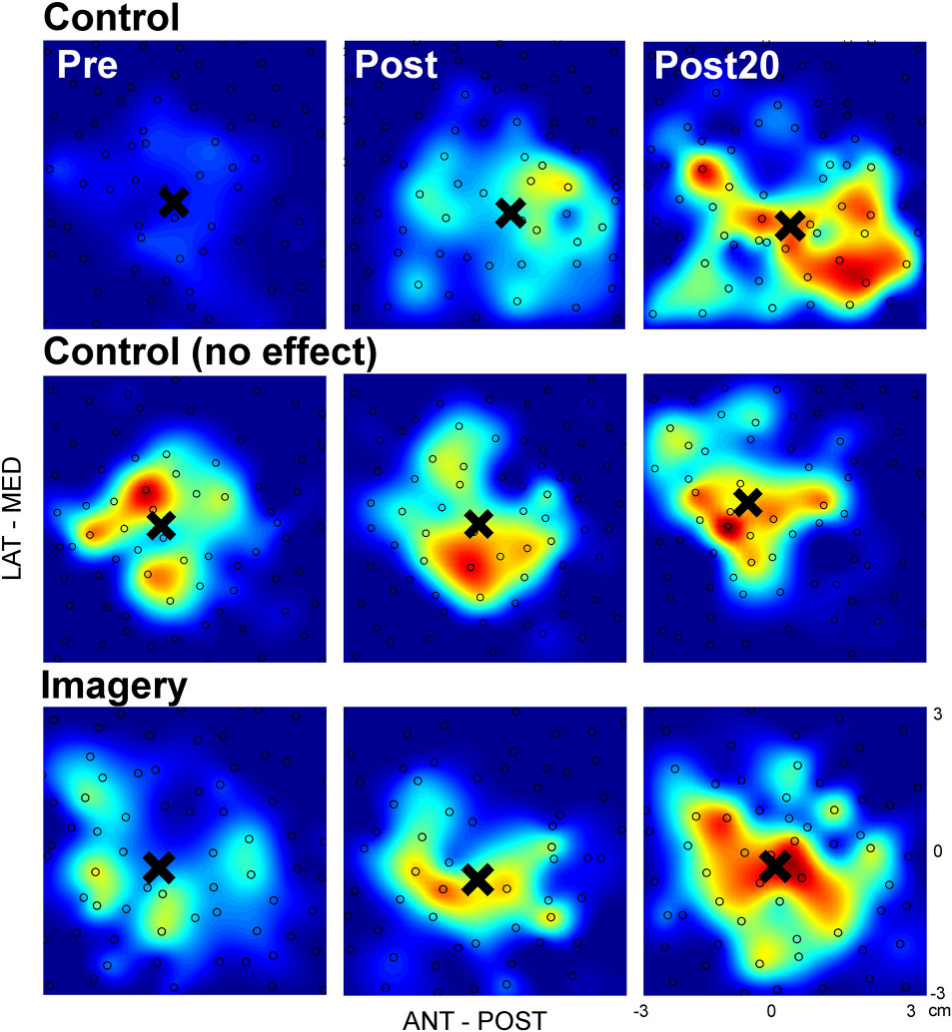
TMS maps before and after mirror training for representative participants in both the control and imagery group. The size of the approximated MEP_pp_ is indicated by the color, with blue representing no or small MEPs and red representing the greatest MEPs. In each map the black open circles mark one stimulation and the black cross highlights the COG (**×**). **(Top)**: Participant from the control group with an increased map area from before to 20 min after training; **(Middle)** Participant from the control group with no change in map area from before to 20 min after training; **(Bottom)** Participant from the imagery group with an increased map area from before to 20 min after training. No systematic shifts in COG can be observed for any participant in these examples.

Figure 2 shows single participant data from both the control and imagery group. In this figure, one TMS map is shown for each time point and three different participants, two from the control group (top and middle row), and one from the imagery group (bottom row). The data from the control (top row) and imagery group (bottom row) represents a positive responder, with an increase in map area as evident from the greater presence of larger MEPs (more red). In contrast, also a non-responder (middle row) is shown, with no change in map area. In all cases, the COG (highlighted by the black cross) is unaffected by the training.

**Figure 3:**
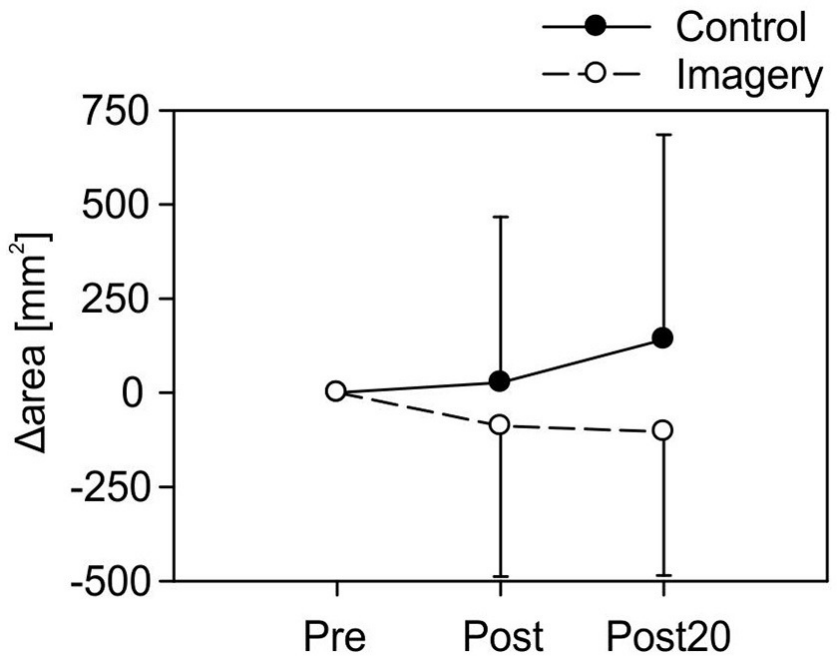
Group data for the map area for both the control and imagery group at all-time points (Mean ± 1 SD). No significant differences were found from before to after mirror training for any group.

A fixed one sample *t*-test for Δarea, compared to 0, revealed no effect of mirror training for any time point or group following training [Control group: Post: *t*_(19)_ = 0.27, *P* = 0.79; Post20: *t*_(19)_ = 1.16, *P* = 0.26; Imagery group: Post: *t*_(19)_ = −0.99, *P* = 0.34; Post20: *t*_(19)_ = −1.22, *P* = 0.24]. Comparing map area following training between the groups, a two-way repeated measures ANOVA (within factor: time point, between factor: group) revealed a non-significant effect [*F*_(38, 1)_ = 2.01, *P* = 0.17]. Moreover, no effect for the map area was found for training [*F*_(38, 1)_ = 0.67, *P* = 0.42] or the training × group interaction [*F*_(38, 1)_ = 1.15, *P* = 0.29]. Group data for map area is shown in Figure 3.

No effect of mirror training was found on the COG for the control group [Δx: Post: *t*_(19)_ = 0.68, *P* = 0.50; Post20: *t*_(19)_ = 0.26, *P* = 0.80; Δy: Post: *t*_(19)_ = 1.06, *P* = 0.31; Post20: *t*_(19)_ = 1.47, *P* = 0.16] or imagery group [Δx: Post: *t*_(19)_ = −0.09, *P* = 0.93; Post20: *t*_(19)_ = −0.69, *P* = 0.50; Δy: Post: *t*_(19)_ = 1.40, *P* = 0.18; Post20: *t*_(19)_ = 1.12, *P* = 0.28]. In addition, a two-way repeated measures ANOVA revealed no differences between the groups, time points after training or group × time point interaction [Δx: time point: *F*_(38, 1)_ = 1.07, *P* = 0.31, group: *F*_(38, 1)_ = 0.42, *P* = 0.52, group × time point: *F*_(38, 1)_ = 0.54, *P* = 0.70; Δy: time point: *F*_(38, 1)_ = 0.85, *P* = 0.36, group: *F*_(38, 1)_ = 0.47, *P* = 0.59, group × time point: *F*_(38, 1)_ = 1.74, *P* = 0.19].

**Figure 4:**
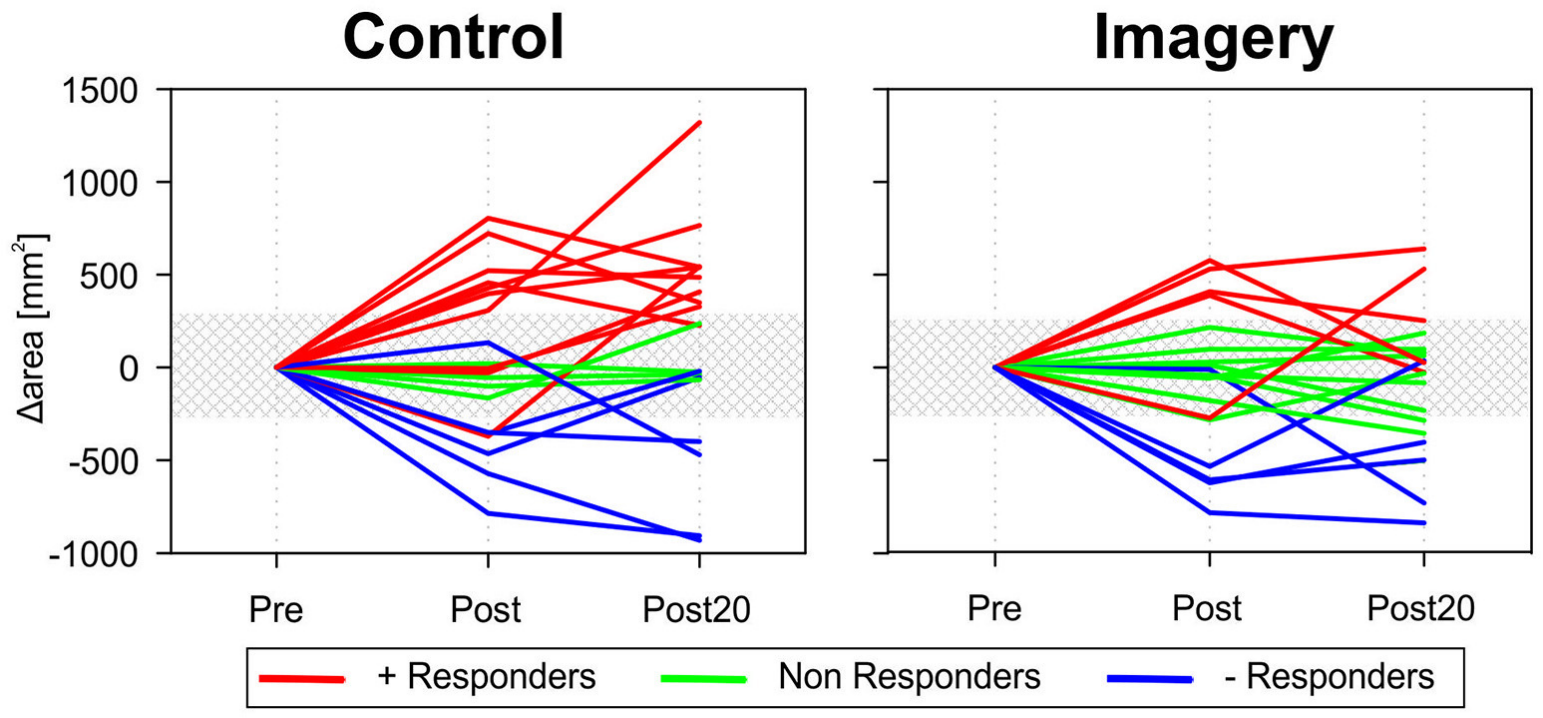
Map area before and after mirror training for all participants individually in both groups. Variability in the response to the training can be observed in both the control and imagery group. Participants were either classified as a positive responder (+ Responder − red lines) or negative responder (− Responder − blue lines) when the increase or decrease in map area was greater than the baseline variability (shaded area). For the control group, 10 participants were classified as positive responder, 6 as negative responders, and 4 as non-responder. In contrast in the imagery group, there were 5 positive, 5 negative, and 10 non-responders.

These inconclusive results can be partly explained by the variability associated with the mirror training. Figure 4 shows the changes in TMS map area following mirror training for each participant in the control and imagery group. Participants show either a strong increase, strong decrease, or no change in the map area. Classifying participants as positive-, negative-, or non-responder based on the change in map area revealed that for the control group, 10 participants were classified as positive responder, 6 as negative responders and 4 as non-responder. In contrast in the imagery group, there were 5 positive, 5 negative, and 10 non-responders.

### Post training evaluation questionnaire

Participants reported that, on average, they were engaged 80% of the time during the mirror training, with no difference found between the groups (control: 80 ± 12%; imagery: 78 ± 14%: *t* = −0.97, *P* = 0.34). Ratings for all other questions can be found in Table 1.

**Table 1:** Results of the task evaluation questionnaire for both groups (^**^indicates a significant difference between the groups).

	**Control (*n* = 20)**	**Imagery (*n* = 20)**
	**mean ± *SD***	**mean ± *SD***
**Did you …**.		
… feel your imagery improved? *(1: not at all, 7: considerably)*	5.45 ± 1.10	5.80 ± 0.77
… image as instructed and practiced? ^**^(1: not at all, 7: exactly)	5.65 ± 0.67	6.20 ± 0.70
… notice any movement in the non-dominant hand? *(1:not at all, 7: full grasping)*	3.70 ± 1.08	3.32 ± 1.25
**How …**		
… easy to image the feelings? *(1: very hard, 7: very easy)*	5.00 ± 1.41	5.20 ± 1.01
… clear and vivid was the imagery? *(1: no image at all, 7: perfectly clear/vivid)*	5.40 ± 0.94	5.45 ± 0.76

*All questions were rated on a 7-point scale. A significant difference was found for the ability to image as instructed and practiced, showing successfulness of the applied imagery training. No other significant differences were found*.

For most participants imagery did get better throughout the eight training blocks, and they fulfilled the task as instructed and practiced. Participants in the imagery group rated the question if they had imaged as instructed and practiced significantly higher than the control group (*t* = −2.55, *P* = 0.02). For the other questions no significant differences were found between the groups. In general, participants reported finding it easy to attribute the feelings of a grasping movement to the non-dominant passive hand, with the image being fairly clear and vivid.

### Predicting factors of response to mirror training

A 2w-MANCOVA revealed no significant main effects for either group [*F*_(2, 23)_ = 0.66, *P* = 0.53; Wilk's Λ = 0.95], Time or the day [*F*_(6, 46)_ = 1.90, *P* = 0.10; Wilk's Λ = 0.64] or group × time of the day interaction [*F*_(6, 46)_ = 1.55, *P* = 0.18; Wilk's Λ = 0.69]. Two of the eight included covariates were found significantly related to the TMS map area: vividness of the imagery [*F*_(2, 23)_ = 10.01, *P* < 0.01; Wilk's Λ = 0.54] and internal imagery ability [*F*_(2, 23)_ = 3.85, *P* = 0.04; Wilk's Λ = 0.75]. Further analysis of the parameters of the estimates regression equations showed that participants better at internal imagery and rating their imagery as being very vivid and clear tended to have a smaller increase (or greater decrease) in TMS map area.

## Discussion

This study aimed to determine if a single session of LSRT in addition to mirror training would induce significantly greater changes in TMS map area than mirror training alone. Based on the potential link between motor imagery and mirror training, we hypothesized that mirror training augmented by a brief period of motor imagery training would exhibit increased TMS map area compared with mirror training alone.

This study presents the somewhat surprising finding that neither mirror training alone nor motor imagery augmented mirror training produce changes in TMS map area. This contrasts the observations of Nojima et al. (2012) who observed increased MEP amplitude following mirror training in healthy participants. Other studies also report increases in MEP amplitude (Garry et al., 2005; Fukumura et al., 2007; Funase et al., 2007) and reduction in intracortical inhibition (Zult et al., 2015) associated with the passive hand behind the mirror but assessed MEP amplitude whilst the mirror training was performed. Similar increases in MEP amplitude (Fadiga et al., 1995; Kasai et al., 1997; Rossini et al., 1999; Strafella and Paus, 2000) or TMS map area (Pascual-Leone et al., 1995; Cicinelli et al., 2006) have been reported when motor imagery and action observation are performed. Therefore, motor imagery was expected to enhance mirror training induced changes in TMS map area.

### Drivers of plasticity: effect on changes in TMS map area following mirror training

Use-dependent plasticity within the central nervous system is driven by the type of motor task performed (commonly called task-specificity), number of repetitions and time the task is performed, level of skill of the task (challenge), and motivation (Nudo et al., 1996; Plautz et al., 2000; Sanes and Donoghue, 2000). In the present study, repetitive hand opening and closing was chosen in order to replicate the type of mirror training that might be used in a motor rehabilitation setting. This simple training task is in line with a repetitive finger tapping task the adopted by Avanzino et al. (2014), but requires less skill than the similar study of Nojima et al. (2012) in which participants performed a challenging ball rotation task. Nojima et al. reported a significant increase in MEP amplitude after only 5 min of training, whereas Avanzino et al. (2014) observed no change in MEP amplitude of the non-trained hand following 10 min of finger tapping. Furthermore, greater changes in MEP amplitude have been observed for a target directed grasping movement compared with a grasping movement alone (Enticott et al., 2010). This suggests within a single training session, the skill/challenge element of the training might be a stronger driver of an increase in MEP amplitude than the number of repetitions. We conducted pilot testing during which we explored the benefits of introducing an object in mirror training similar to Nojima et al; however, our participants reported greater difficulty for the motor imagery component of the task when handling an object. The increased proprioceptive input associated with handling an object may have created an internal conflict between visual and proprioceptive information.

Repetitive use alone has led to changes in MEP amplitude. For example, Byblow et al. (2012) demonstrated 20 min of repetitive passive wrist flexion and extension driven by a robotic manipulator results in an increase in MEP amplitude, when a mirror symmetric movement was made with the other wrist. MEP amplitude may also be found as changes in TMS map area. Pascual-Leone et al. (1995) used a control group that performed a simple one-handed piano exercise for 2 h a day and demonstrated an increase in TMS map area after a single session of training. Therefore, although the grasping movement employed in the present study required little skill, small differences in TMS map area might still have been expected when the imagery training was employed. The training time in the present study was aimed to maximize number of repetitions whilst still maintaining the participant's concentration on the task. In the present study, we used 60% more training compared with Avanzino et al. (2014) but we still did not see a change in CSE. Nevertheless, the possibility cannot be excluded that additional training would provide a different result. It is unlikely that an increased session time would increase CSE, because the participants motivation and attendance to the task would wane. However, it is possible that we would have seen a different result had the participants been exposed to several training sessions over a few days as was done by Pascual-Leone et al. (1995). Importantly, it cannot be concluded from the results of the present study that changes in CSE, plasticity and functional change would not be observed in hemiparetic patients exposed to a similar task, because the challenge to such patients, their capacity for use-dependent plasticity, and the training time would be far greater than the healthy people who participated here.

An additional driver of plasticity relating to mirror training and motor imagery may be the strategy that participants adopt to perform the mirror training when they have difficulties to remain focused and receive conflicting sensory information (proprioceptive and visual). Emphasis could be put either on observing or imaging. It is likely participants will unconsciously determine strategy based on the instructions provided. In this study, participants were explicitly instructed to both feel (kinaesthetic motor imagery) and see (action observation and visual imagery) the hand behind the mirror making the repetitive grasping movement. Participants in the imagery group received 10–15 min of imagery training and, therefore, it is likely that these participants will have adopted an imagery focused strategy during mirror training. In contrast, as no specific practice was performed, participants in the control group could only rely on their interpretation of the instructions. Participants may have adopted a strategy that was easiest for them, either focussing on feeling or seeing the hand move. Recently, Ferron and Tremblay (2017) already suggested that the use of a more visual strategy reduces the need for M1 involvement possibly explaining the lack of change in TMS map area. In addition, since motor imagery is an active process which, especially when performed repetitively over 16 min, is mentally demanding, participants may have unconsciously shifted strategy throughout training when having difficulties to remain focussed. As the instructions given to the participants are rarely reported, it is difficult to compare the outcome of different studies based on the likely adopted strategy.

Whereas different studies report changes in MEP amplitude and TMS map area following mirror training and motor imagery, these studies assessed CSE before and directly after training. Pascual-Leone et al. (1995) demonstrated consolidation is important and changes in TMS map area can only be observed 20 min after training. A subsequent review from Pascual-Leone et al. (2005) mentioned 20–30 min of consolidation time, suggesting a longer consolidation period may be required. Therefore, in this study CSE was assessed both directly as well as 20 min after the mirror training. TMS map area did not change from directly to 20 min after the training and there was no consistent trend indicating increasing TMS map area with time.

### Variability associated with plasticity and motor imagery ability

Although the used methods or adopted mirror training strategy by participants in this study might have affected the outcome, it is not uncommon for use-dependent and non-invasive brain stimulation induced plasticity to only be present in a subset of the participants tested (Muller-Dahlhaus et al., 2008; Hamada et al., 2013; Lopez-Alonso et al., 2014; Wiethoff et al., 2014). In the mirror group, 10 participants responded with an increased TMS map area (50%), which matches the earlier reported response rates. However, only 5 out of 20 (25%) of the participants responded with an increased TMS map area when the imagery training was introduced.

Both attention and the possible internal conflict between visual and proprioceptive information may well have mediated this reduced response rate. The possible internal conflict between visual and proprioceptive information may explain why the change in map area was smaller for participants with better internal imagery ability and for those that the imagery was more clear and vivid. This does suggest that in this study imagery training has worked counterproductive, as better imagery ability leads to smaller changes in TMS map area. It is not unthinkable that the better imagery ability has drawn attention of the participant to the existing conflict between proprioceptive and visual feedback, reducing the effect of the mirror training. Hence, the existing conflict may also have affected attention, which has been shown to affect changes in MEP amplitude and primary motor cortex activation (Binkofski et al., 2002; Rosenkranz and Rothwell, 2006; Kamke et al., 2012). Direction of attention, combined with the participant's imagery ability, has been shown lately to affect motor learning rate during learning (Sakurada et al., 2016). Participants good at KI benefitted from directing attention internally, whilst those good in visual imagery found an external focus advantageous. Because of the between subject design and not explicitly specifying strategy or were to direct attention, it cannot be excluded that the variable changes in TMS map area have been a result of the different participants tested in each group. However, it has to be stressed that, in general, direction of attention is not something that is specified in the task instruction for mirror training or, at least, this is not reported in literature.

To enable better quantification of whether imagery ability affects plasticity it would be beneficial for future studies to incorporate several different measures of imagery ability as suggested by Guillot and Collet (2005) and Saimpont et al. (2015). These involve for example mental chronometry, skin conductance measurement and mental rotation tasks. The assessment of imagery ability using the MIQ-3 questionnaire is subjective and did not allow us to exclude any participants based on their imagery ability. Based on the MIQ-3 score, most of the participants in our study were good at imagery. As imagery ability is known to affect changes in MEP amplitude and neuronal activation (Guillot et al., 2008; Williams et al., 2012), additional measures might allow for a better insight in participants imagery ability and ultimately reduce the variability in the response to mirror training.

The inclusion of extra measures to quantify imagery ability may allow to create a better understanding of how imagery ability, changes in CSE and motor learning are related. For example, the non-significant trend in this study that participants better at imagery tended to have a smaller increase (or decrease) in map area is interesting, but the current data set is not big enough to draw any firm conclusions. To strengthen these findings a subgroup analysis could have been used. This was, however, deemed unfeasible, given the the low number of participants that would end up in a group. Future studies may need to pre-screen participants based on imagery ability to create a homogenous sample of participants for different levels of imagery ability that allows to assess its's effect further.

## Conclusion

The aim of this study was to explore whether mirror training augmented with motor imagery training would significantly increase changes in TMS map area compared to mirror training alone. The results show a single session of 16 min of mirror training (with or without imagery training) does not lead to significant changes in TMS map area. Whereas, no changes in map area were observed following either intervention in the present study, this may be a result of the specific experimental conditions and different imagery training may still benefit mirror training when the study is performed in a different way (e.g., longer mirror and/or imagery training). Moreover, these results cannot be extended to therapeutic practice involving mirror training or motor imagery as there is greater scope for change in patients with acquired brain injury compared with healthy participant who already have very good motor ability with this task.

This study performed in a patient group, e.g., stroke survivors, rather than healthy participants, might reveal that imagery training can be an important addition to mirror training. In stroke patients many challenges faced when training healthy people are not present, e.g., internal conflict will not be a problem as they are explicitly instructed to perform the training task bilaterally (Stoykov and Corcos, 2009). Moreover, imagery and mirror training may need to be combined on a longer time scale to allow the imagery to benefit the mirror training. The short duration of training performed here may well have been insufficient to observe changes in CSE, and multiple training sessions may be required to exploit the benefit of imagery training. As there are many methodological confounding factors it is important that authors clarify their methodology, e.g., task instruction, so studies can be better compared. This might be a first step into further disentangling the role of imagery and action observation in mirror training so that mirror training can be further tailored to the individual according to their abilities.

## Author contributions

MvdR: conducted the whole study and drafted the manuscript; MvdR and MG: contributed in problem identification; MvdR: collected the experimental data; MvdR and MG: participated in editing the manuscript.

### Conflict of interest statement

The authors declare that the research was conducted in the absence of any commercial or financial relationships that could be construed as a potential conflict of interest.
